# Cell differentiation is disrupted by MYO5B loss through Wnt/Notch imbalance

**DOI:** 10.1172/jci.insight.150416

**Published:** 2021-08-23

**Authors:** Izumi Kaji, Joseph T. Roland, Sudiksha Rathan-Kumar, Amy C. Engevik, Andreanna Burman, Anna E. Goldstein, Masahiko Watanabe, James R. Goldenring

**Affiliations:** 1 Section of Surgical Sciences and; 2 Epithelial Biology Center, Vanderbilt University Medical Center, Nashville, Tennessee, USA.; 3 Department of Cell and Developmental Biology, Vanderbilt University School of Medicine, Nashville, Tennessee, USA.; 4 Department of Anatomy, Faculty of Medicine, Hokkaido University, Sapporo, Japan.; 5 Nashville VA Medical Center, Nashville, Tennessee, USA.

**Keywords:** Gastroenterology, Epithelial transport of ions and water, Genetic diseases, Protein traffic

## Abstract

Functional loss of myosin Vb (MYO5B) induces a variety of deficits in intestinal epithelial cell function and causes a congenital diarrheal disorder, microvillus inclusion disease (MVID). The impact of MYO5B loss on differentiated cell lineage choice has not been investigated. We quantified the populations of differentiated epithelial cells in tamoxifen-induced, epithelial cell–specific MYO5B-knockout (*VilCre^ERT2^ Myo5b*^fl*/*fl^) mice utilizing digital image analysis. Consistent with our RNA-sequencing data, MYO5B loss induced a reduction in tuft cells in vivo and in organoid cultures. Paneth cells were significantly increased by MYO5B deficiency along with expansion of the progenitor cell zone. We further investigated the effect of lysophosphatidic acid (LPA) signaling on epithelial cell differentiation. Intraperitoneal LPA significantly increased tuft cell populations in both control and MYO5B-knockout mice. Transcripts for Wnt ligands were significantly downregulated by MYO5B loss in intestinal epithelial cells, whereas Notch signaling molecules were unchanged. Additionally, treatment with the Notch inhibitor dibenzazepine (DBZ) restored the populations of secretory cells, suggesting that the Notch pathway is maintained in MYO5B-deficient intestine. MYO5B loss likely impairs progenitor cell differentiation in the small intestine in vivo and in vitro, partially mediated by Wnt/Notch imbalance. Notch inhibition and/or LPA treatment may represent an effective therapeutic approach for treatment of MVID.

## Introduction

Myosin Vb (MYO5B) is an essential motor protein for apical membrane protein trafficking, and its inhibition through truncation or missense mutations causes the congenital diarrheal disorder microvillus inclusion disease (MVID) (1, 2). Several animal models and patients with MVID with MYO5B mutations demonstrate mislocalization of a variety of enterocyte membrane transporters and enzymes, resulting in malabsorption (3–6). We have reported that germline deletion of MYO5B in mice causes expanded crypt length and increased Paneth cell numbers (4). Recently, we reported that tamoxifen-induced, intestine-specific MYO5B deficiency in *VilCre^ERT2^* *Myo5b^fl/fl^* mice demonstrates hyperproliferation and villus blunting. Treatment of *VilCre^ERT2^* *Myo5b^fl/fl^* mice with a bioactive phospholipid, lysophosphatidic acid (LPA), partially restored the villus structure, brush border height, and apical localization of nutrient transporters in the small intestine (7). Based on these observations, we hypothesized that MYO5B loss causes differentiation deficits in intestinal epithelial cells and that LPA supplementation may promote epithelial differentiation and microvillus maturation. The function of MYO5B and LPA signaling in differentiation of rare epithelial cell populations, however, has not been investigated.

Secretory cell lineages in the small intestine are rare (<5%) populations and include 4 subsets: Paneth, goblet, enteroendocrine, and tuft (also known as brush) cells. Paneth cells are localized at the base of crypts and secrete antibacterial components into the intestinal lumen and support the stem cell niche (8). Goblet cells are scattered along the crypt-to-villus axis and secrete mucin 2 (MUC2) together with trefoil factor 3 (TFF3) for mucosal protection. Enteroendocrine cells produce gut hormones, including neuropeptides and amines, and regulate intestinal function as well as appetite. Tuft cells are considered chemosensory cells based on the expression of taste cell–specific molecules, such as transient receptor potential cation subfamily M member 5 (TRPM5) and gustducin, and the transcription factor for sensory cell lineage, POU class 2 homeobox 3 (POU2F3) (9, 10). Mouse intestinal tuft cells can be stained with neuronal and arachidonic acid signaling markers, including doublecortin like kinase (DCLK1), choline acetyl transferase (ChAT), and cyclooxygenase 1, a product of the gene prostaglandin-endoperoxide synthase 1 (*Ptgs1*). A decreased tuft cell population has been identified in pediatric patients with celiac disease and duodenitis, consistent with the nutrient-sensory function of tuft cells and malabsorption symptoms in intestinal disorders (11). RNA-sequencing data for isolated jejunal epithelial cells from *VilCre^ERT2^* *Myo5b^fl/fl^* mice demonstrate that transcriptional signatures of each type of differentiated cell and stem cell are significantly altered by MYO5B loss (Gene Expression Omnibus data set ID: GSE139302) (7). In particular, tuft cell–specific transcription factors, *Pou2f3* (log_2_ fold change: –2.3) and *Spib* (log_2_ –1.7), and mature tuft cell markers, *Chat* (log_2_ –2.5), *Ptgs1* (log_2_ –2.2), *Dclk1* (log_2_ –1.2), *Plcb2* (log_2_ –1.7), *Trpm5* (log_2_ –1.3), *Gfi1b* (log_2_ –1.0), and *Sucnr1* (log_2_ –1.0), were notably downregulated by MYO5B deficiency, 4 days after Cre recombinase induction (7). Therefore, in the present study, we performed quantitative analysis of immunostaining for each type of epithelial cell in the whole small intestine of *VilCre^ERT2^* *Myo5b^fl/fl^* mice utilizing digital image analysis. MYO5B loss significantly decreased the frequency of goblet cells and tuft cells, whereas Paneth cells were increased along with an expanded progenitor cell zone. Expressions of transient amplifying cell markers were differentially altered by MYO5B deficiency, and epithelial cell–derived Wnt ligands were significantly downregulated in MYO5B-knockout intestine. Notch signaling was maintained in MYO5B-knockout intestine at comparable levels to that in control mice. The altered balance of Wnt/Notch signaling pathways may explain at least some of the alterations in stem cell signatures and differentiation toward sensory cells following MYO5B loss.

## Results

### Loss of MYO5B results in decreased intestinal tuft cell numbers, and intraperitoneal LPA reestablishes tuft cell frequency.

**Figure 1:**
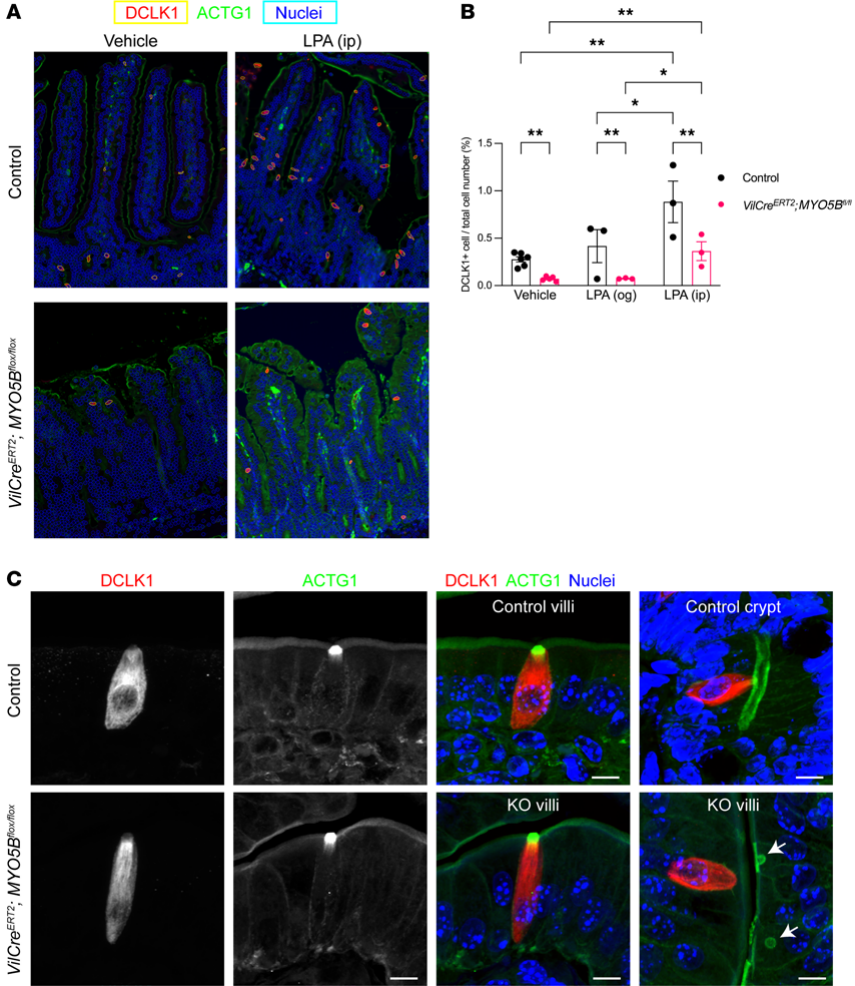
Changes in frequency of intestinal tuft cells by MYO5B loss and ip administration of LPA. (**A**) Representative overlaid images of immunostaining and digitally identified cells. Whole-slide images of small intestine were analyzed. All nuclei were identified by Hoechst signals (shown in blue) and circled by light blue, and DCLK1^+^ tuft cells (shown in red) were defined with yellow circles. Original magnification is 20×. (**B**) Quantification of tuft cell frequency. Total cell number was counted by nuclei. Mean ± SD. Each data point represents a value from individual mouse. *P < 0.05, **P < 0.01 analyzed by 2-way ANOVA with Tukey’s multiple comparison. *n* = 3–6 mice per group. (**C**) *Z*-stack confocal images of DCLK1 (red) and ACTG1 (green) in tuft cells in control and induced MYO5B-deficient mouse jejunum. Vertical sections of tuft cell show dense ACTG1^+^ microtubule structure in both villi and crypts of control intestine. Villus tuft cells were present in MYO5B-deficient intestine with similar morphologies to those in controls. Arrows: microvillus inclusions in MYO5B-deficient enterocytes. Scale bars: 10 μm. ACTG1, actin gamma 1.

Intestinal tuft cells and enteroendocrine cells are distinct populations of chemosensory cells that detect luminal and/or circulating metabolites and regulate gastrointestinal function (12, 13). Using digital image analysis tools, we quantified the populations of DCLK1^+^ tuft cells and chromogranin A (ChgA^+^) enteroendocrine cells in the entire small intestine (duodenum, jejunum, and ileum) from tamoxifen-induced MYO5B-knockout mice and tamoxifen-injected control littermates. Figure 1A shows the output images of digitally identified tuft cells and nuclei that were overlaid on immunostaining images by Cell Profiler. The frequency of DCLK1^+^ cells was 80% lower in MYO5B-deficient intestine than control, particularly in the crypts, indicating that tuft cell differentiation was inhibited by MYO5B loss (Figure 1B). LPA administration by intraperitoneal (ip) injection for 4 days, but not by oral gavage (og), significantly increased tuft cell density both in control and in MYO5B-deficient mice, suggesting that a differentiation pathway toward the tuft cell lineage was activated by systemic LPA receptor activation (Figure 1, A and B). Tuft cell morphologies were compared between control and MYO5B-deficient jejunum in *Z*-stack imaging using confocal microscopy. DCLK1^+^ tuft cells possess prominent ACTG1-containing apical microvilli, which are denser and longer compared with enterocyte brush borders (Figure 1C). In control intestine, the ACTG1^+^ tuft structure was found in tuft cells both in villi and in crypts. This tuft cell–specific ACTG1 staining pattern is similar to that of acetylated tubulin and F-actin, which is stained by phalloidin in frozen sections (14). MYO5B-deficient intestine had similar structures in tuft cells in the villi (Figure 1C). Consistent with our previous report (7), the brush borders in MYO5B-deficient enterocytes around tuft cells were thinner than in control intestine, and microvillus inclusions were occasionally observed in enterocytes in the villi (Figure 1C), confirming the induction of MYO5B loss by tamoxifen injection. DCLK1^+^ cells were rarely found in the crypts of MYO5B-deficient intestine, suggesting that MYO5B loss inhibited tuft cell differentiation. Previous lineage tracing studies in *Dclk1-Cre* reporter mice demonstrated that DCLK1-expressing cells live up to 7 days (15). Thus, the villus tuft cells in the induced MYO5B-knockout mice likely survive for 4 days after Cre-recombinase induction.

**Figure 2:**
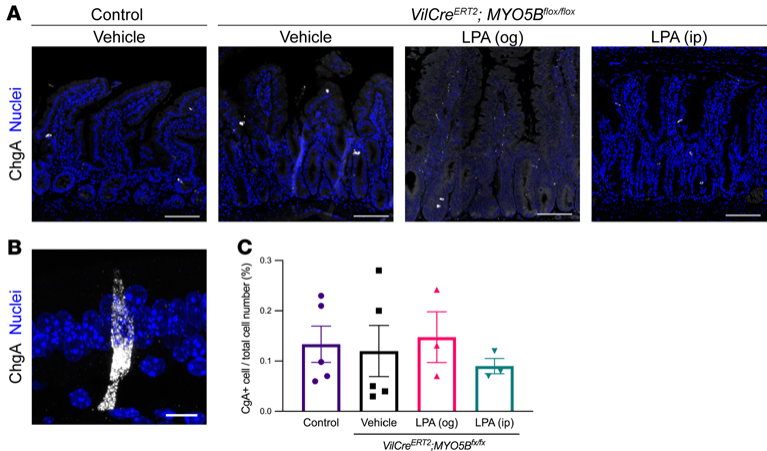
Lack of changes in the frequency of enteroendocrine cells in MYO5B-deficient intestine. (**A**) Representative immunostaining for chromogranin A (ChgA) in jejunal sections from control and induced MYO5B-knockout mice 4 days after tamoxifen injection. ChgA^+^ cells were scattered throughout the crypt-to-villus axis in all treatment groups. Scale bar: 100 μm. (**B**) Representative high-magnification image of ChgA immunoreactivity. ChgA^+^ secretory granules were distributed in the cytosol. Scale bar: 10 μm. (**C**) Quantification of enteroendocrine cells in whole small intestine. Mean ± SD. Each data point represents a value from an individual mouse. No significant difference was detected by 1-way ANOVA.

ChgA is a secretory granule protein that is specifically produced by enteroendocrine cells in the intestine. The frequency and morphology of ChgA^+^ cells in intestinal sections were similar between control and MYO5B-knockout mice (Figure 2). Some enteroendocrine cells in the small intestine may make direct contacts with nerve terminals and live longer than other types of intestinal epithelial cells (16). Since MYO5B loss induces debilitating malabsorption and secretory diarrhea in *Villin-Cre^ERT2^* *Myo5b^fl/fl^* mice within 5 days after tamoxifen injection, this model is not ideal to address the effect of MYO5B loss on the differentiation of long-lived enteroendocrine cells.

### TFF3-producing goblet cells are decreased by MYO5B loss.

**Figure 3:**
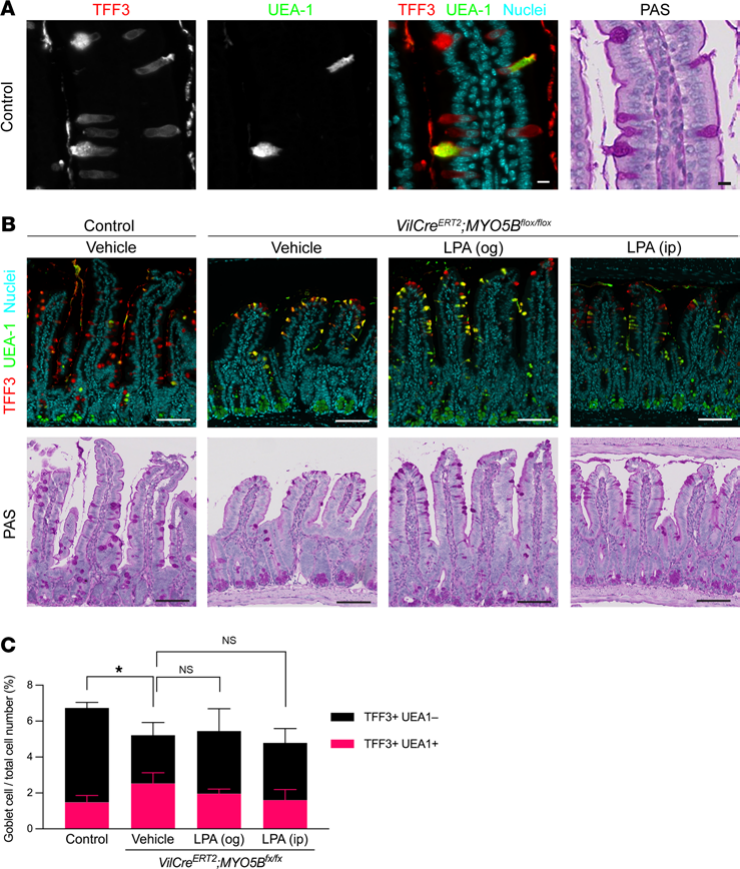
Decrease in frequency of goblet cells in MYO5B-deficient mouse small intestine. (**A**) Colocalization of TFF3-immunoreactivity with PAS staining in goblet cells of control mouse intestine. TFF3 staining was detected in luminal mucus and in the cytosol of goblet cells similar to the intense red staining by PAS. UEA-1 staining was found in a subset of mucus granules. Scale bars: 10 μm. (**B**) Representative staining for goblet cells in jejunum from control and tamoxifen-induced MYO5B-knockout mice after 4-day treatment with vehicle, og LPA, or ip LPA. Scale bars: 100 μm. (**C**) Quantification of UEA-1– and/or TFF3-immunoreactive goblet cells in whole small intestine using digital image analysis. *n* = 4 mice per group. Mean ± SD. *P < 0.05 in total goblet cell numbers analyzed by 1-way ANOVA with Dunnett’s multiple comparison.

MYO5B loss significantly decreases the transcription factor NF-κB subunit 2 (*Nfkb2*) (log_2_ –1.6) (7), which is predominantly expressed in goblet cells (17). TFF3 and a fucose-binding lectin, *Ulex europaeus* agglutinin 1 (UEA-1), have been widely used to identify intestinal mucus-secreting intestinal goblet cells. In this study, we observed a substantial number of TFF3^+^ cells were not stained by UEA-1, particularly in tips of villi in control intestine. MUC2 is also secreted from intestinal goblet cells, and only 50% of MUC2^+^ cells are stained with UEA-1 in mouse ileum (18). To confirm whether TFF3^+^UEA-1^–^ cells produce mucin, period acid–Schiff (PAS) staining was performed on the same slides after imaging for TFF3 and UEA-1 (Figure 3A). UEA-1 signals were detected in granules of a subset of TFF3-immunoreactive goblet cells and Paneth cells, while TFF3 signals labeled the entire cytosol of goblet cells (Figure 3A). Since all TFF3^+^ cells with or without UEA-1 were stained with PAS, TFF3 was used as a general goblet cell marker for quantification. In control mouse intestine, TFF3^+^ and PAS-stained goblet cells were scattered from the top part of crypts through the villi, and the TFF3^+^UEA-1^+^ cells were limited to approximately 20% of total goblet cells (Figure 3, B and C). Tamoxifen-induced MYO5B-knockout mice demonstrated significantly fewer goblet cells, which accumulated at the tips of villi, suggesting that the differentiation of goblet cells was inhibited by MYO5B loss (Figure 3, B and C). LPA treatment of MYO5B-deficient mice did not significantly increase the frequency of goblet cells.

### MYO5B loss increases Paneth cells and olfactomedin 4–expressing cells along with hyperproliferation.

**Figure 4:**
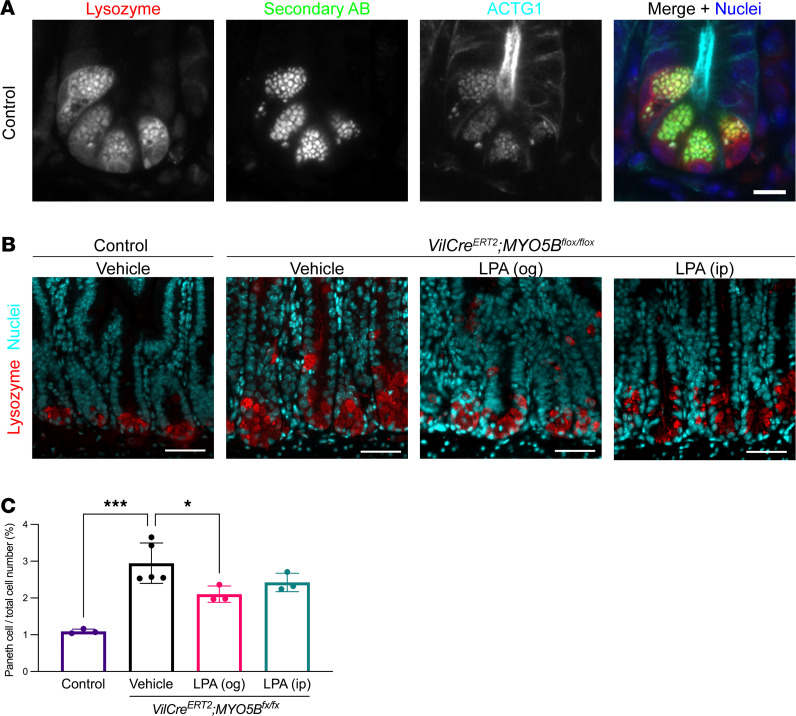
Increased Paneth cells in MYO5B-deficient small intestine. (**A**) Colocalization of lysozyme and autofluorescence in apical granules of Paneth cells. Scale bar: 10 μm. (**B**) Representative immunostaining for lysozyme in jejunum from control and induced MYO5B-knockout mice 4 days after tamoxifen injection. Some Paneth cells in MYO5B-knockout mice migrated toward villi in addition to the crypt bottom. Scale bars: 100 μm. (**C**) Quantification of Paneth cell frequency using digital image analysis. Mean ± SD. Each data point represents a value from individual mouse. *n* = 3–4 mice per group. *P < 0.05; ***P < 0.001 by 1-way ANOVA with Dunnett’s multiple comparison.

Paneth cells reside in the bottom of crypts next to the leucine rich repeat containing G protein coupled receptor 5–expressing (Lgr5-expressing) stem cells and secrete stem cell niche factors as well as lysozyme into the lumen. In the present study, the secretory granules of Paneth cells demonstrated strong autofluorescence without primary antibody (Figure 4A). Quantification by Cell Profiler of this autofluorescence indicated that Paneth cells were significantly increased in tamoxifen-induced MYO5B-deficient intestine (Figure 4, B and C). Our previous report with neonatal germline MYO5B-knockout mice consistently demonstrated more Paneth cells per crypt than control pups (4). Along with the expansion of crypts and hyperproliferation that was demonstrated by Ki67 staining (7), Paneth cells in *VilCre^ERT2^* *Myo5b^fl/fl^* mice were frequently found in the middle portion of crypts, in addition to the base of crypts (Figure 4B). LPA supplementation by gavage significantly decreased Paneth cell frequency in *VilCre^ERT2^* *Myo5b^fl/fl^* mice (Figure 4).

**Figure 5:**
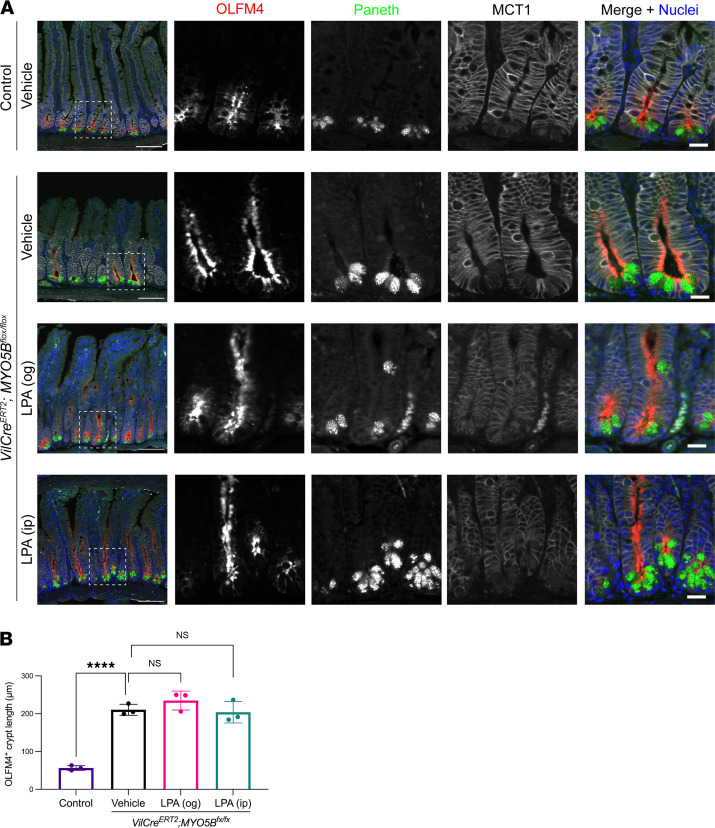
Changes in immunostaining pattern for OLFM4 and MCT1 induced by MYO5B loss. (**A**) OLFM4^+^ stem/progenitor cells were limited to the bottom half of crypts in control intestine. Tamoxifen-induced MYO5B-deficient intestines with or without LPA supplementation demonstrated an extended OLFM4^+^ cell zone along with expanded crypt length. MCT1 was localized on the basolateral membranes of epithelial cells. Control intestine had ubiquitous expression of MCT1 along the crypt-to-villus axis, whereas MYO5B-deficient intestine showed clear MCT1 expression only in the crypt cells. Scale bars: 100 μm in merged images in the left column, 20 μm in the right column. (**B**) OLFM4^+^ crypt length was compared. Each data point represents an average value of 10 measured sites in an individual mouse. *n* = 3 mice per group. ****P < 0.0001 by 1-way ANOVA with Dunnett’s multiple comparison.

MYO5B loss downregulates the transcriptional signature of stem cells, i.e., *Lgr5* (log_2_ –1.4) and achaete-scute family bHLH transcription factor 2 (*Ascl2*) (log_2_ –1.8), but does not alter the expression of the stem cell marker olfactomedin 4 (*Olfm4*) (7), whose expression is independent of Wnt control (19). Three MYO5B-deficient mouse models consistently demonstrate hyperproliferation in the intestinal crypts (4, 7). Monocarboxylate transporter 1 (MCT1) is an essential membrane transporter for supplying short-chain fatty acids and ketone bodies as energy sources in proliferative cells (20). Therefore, next we stained for OLFM4 and MCT1 in the small intestine of *VilCre^ERT2^* *Myo5b^fl/fl^* mice to investigate stem and proliferative cell status. Intense signals for OLFM4 were limited to the apical side in crypt base columnar cells in control intestine, while the OLFM4-expressing crypt region was extended by MYO5B loss with or without LPA supplementation (Figure 5). MCT1 was predominantly expressed in basolateral membranes of control epithelial cells with higher intensity in crypts compared with villi. MYO5B-deficient mouse intestine showed similar MCT1 staining in the crypts, but the MCT1 expression in villus cells was diminished (Figure 5). These observations suggest that stem cell function is impaired by MYO5B loss, possibly because of the suppression of Wnt signaling in progenitor cells.

**Figure 6:**
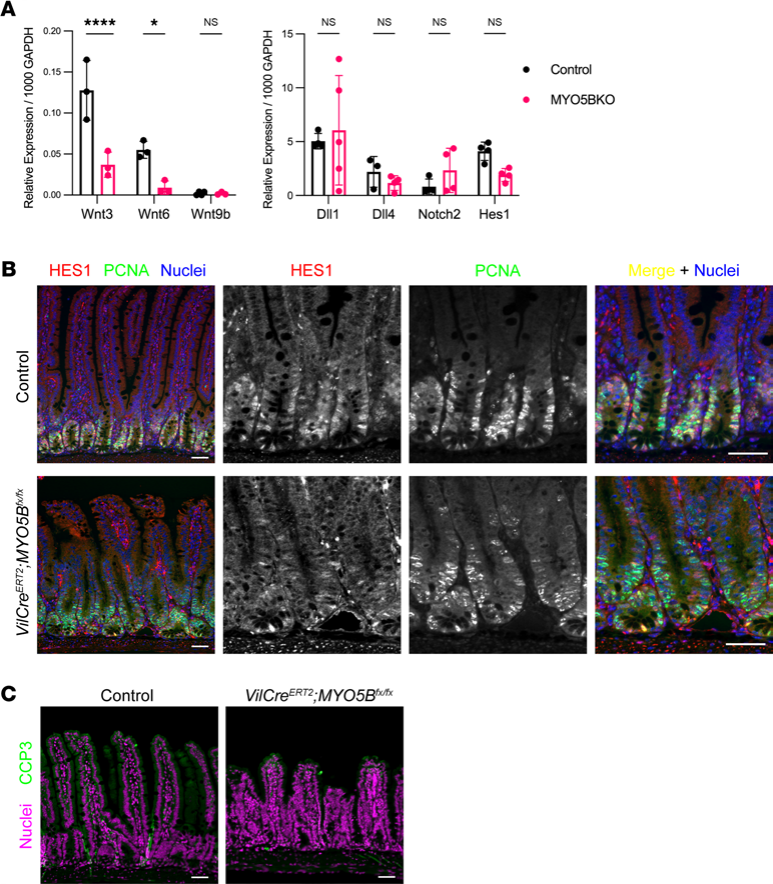
Alteration in the balance of Wnt/Notch signaling induced by MYO5B loss. (**A**) mRNA expressions of Wnt ligands and Notch signaling molecules in isolated epithelial cells were compared between control and tamoxifen-induced MYO5B-deficient mouse jejunum. Mean ± SD. Each data point represents a value from individual mouse. *n* = 3–5 mice per group. *P < 0.05, by 2-way ANOVA with Bonferroni’s post hoc test. (**B**) Immunostaining for a Notch signaling effector, HES1 (red), and a proliferating cell marker, proliferating cell nuclear antigen (PCNA) (green), in mouse jejunum. (**C**) Immunostaining for the apoptosis marker cleaved caspase-3 (CCP3). CCP3^+^ cells were infrequently identified in upper part of villi similarly in control and MYO5B-deficient intestine. Scale bars: 50 μm. Dll, delta like canonical Notch ligand; Hes1, hes family bHLH transcription factor 1.

The gradient of Wnt and Notch signaling along the crypt-villus axis regulates stem cell proliferation and differentiation (21–23). We next examined mRNA expression levels of epithelial cell–derived Wnt ligands and Notch signaling molecules by quantitative reverse transcriptase PCR (qRT-PCR). *Wnt3* and *Wnt6* were significantly decreased in isolated jejunal cells from *VilCre^ERT2^* *Myo5b^fl/fl^* mice compared with control mice (Figure 6A). Our RNA-sequencing data did not detect significant changes but demonstrated slight increases in Notch signaling molecules, including *Atoh1*, *Jag1*, *Dll4*, *Notch1*, *Notch2*, and *Adam10*. By qRT-PCR, we did not detect any significant alteration following MYO5B loss in Notch ligands, Dll1 and Dll4; a Notch receptor, Notch 2; or a Notch effector, Hes1 (Figure 6A). Immunostaining for HES1 in nuclei showed no difference between control and MYO5B-deficient intestine (Figure 6B). LPA treatment of induced *VilCre^ERT2^* *Myo5b^fl/fl^* mice did not alter HES1 immunoreactivity (data not shown). The maintenance of Notch signaling in the presence of reduced Wnt signaling indicates that MYO5B loss may induce Wnt/Notch imbalance and disrupt stem cell renewal and differentiation. Next, we tested if apoptosis is abnormally enhanced by Wnt/Notch imbalance in MYO5B-deficient intestine. Immunostaining for the established apoptosis marker cleaved caspase-3 was occasionally found in the cytoplasm of individual epithelial cells on villus tips at a similar frequency in control and *VilCre^ERT2^* *Myo5b^fl/fl^* mouse small intestine (Figure 6C).

### Tuft cell differentiation in intestinal organoids is suppressed by MYO5B loss in vitro.

**Figure 7:**
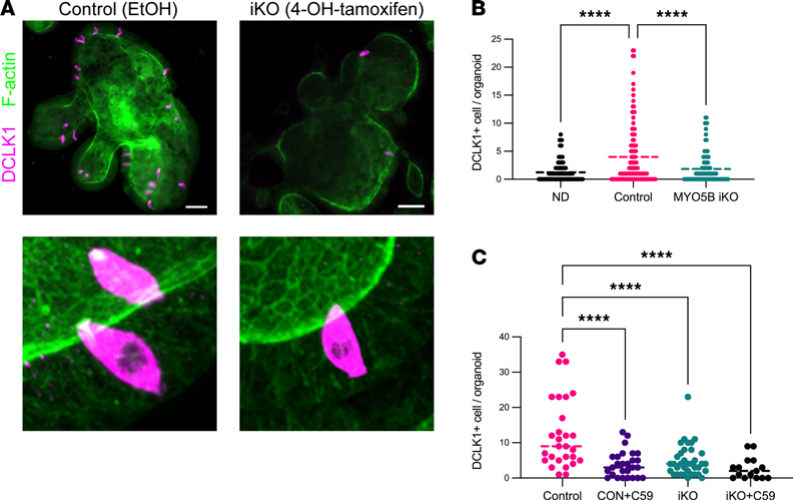
Tuft cell differentiation in *VilCre^ERT2^* *Myo5B^fl/fl^* intestinal organoids. (**A**) Immunostaining for DCLK1 (magenta) and F-actin (green) in whole-mount organoids. MYO5B loss was induced by 4-OH-tamoxifen in vitro (iKO) and control organoids were incubated with ethanol as vehicle. (Inset) Confocal images of F-actin show brush borders and the long microvillus structure in tuft cells. Scale bars: 20 μm. Insets: original magnification is 60×. (**B**) Tuft cell numbers were determined per organoid. ND, nondifferentiated organoids that were cultured for same period in IntestiCult Organoid Growth Medium as differentiated organoids. (**C**) Wnt signaling inhibitor C59 (10 μM) significantly reduced tuft cell differentiation in control organoids to a similar level as in iKO organoids. Each data point represents a value of individual organoid. *n* = 15–36 (**B**) and *n* = 98–133 (**C**) samples from 3 mice per group. ****P < 0.0001 by 1-way ANOVA with Dunnett’s multiple comparison.

To confirm whether tuft cell loss was induced directly in epithelial cells by genetic deletion of MYO5B, we generated organoids from *VilCre^ERT2^* *Myo5b^fl/fl^* mouse jejunum, and Cre recombinase was activated by the addition of 4-OH-tamoxifen in vitro. Forty-eight hours after the withdrawal of Wnt from the media, DCLK1-immunoreactive cells and microvillus inclusions were detected in the induced MYO5B-deficient (iKO) organoids. Figure 7A shows representative immunostaining for DCLK1, which demonstrates the classical flask-like shape of tuft cells in whole-mounted organoids. Tuft cells were distributed in both budding and expanded areas of differentiated control organoids, while iKO organoids showed fewer tuft cells per organoid (Figure 7B). Nondifferentiated organoids had fewer tuft cells than differentiated control organoids after culturing in IntestiCult Organoid Growth Medium without Cre recombinase activation for the identical culture duration as differentiated ones. In high-magnification *Z*-stack projections, phalloidin-positive, dense microvilli were found in tuft cells in control organoids similar to the distinct tuft structures in the native small intestine of mice.

Since we found downregulated expression of Wnt ligands in the MYO5B-deficient mouse intestine (Figure 6), we next tested the effect of the Wnt signaling inhibitor C59 on organoids. The addition of C59 (10 μM) to the differentiation medium significantly reduced tuft cell numbers in control organoids to a similar value to that in MYO5B-iKO organoids (Figure 7C). This result indicates that active Wnt signaling may accelerate tuft cell differentiation and that the control organoids may secrete endogenous Wnt ligands under culture conditions in differentiation medium that lacks exogenous Wnt. Addition of C59 did not decrease tuft cell numbers in MYO5B-iKO organoids, further suggesting that MYO5B-deficient organoids have reduced Wnt source(s) (Figure 7C).

### Acute Notch signaling inhibition improves enterocyte and secretory cell differentiation.

**Figure 8:**
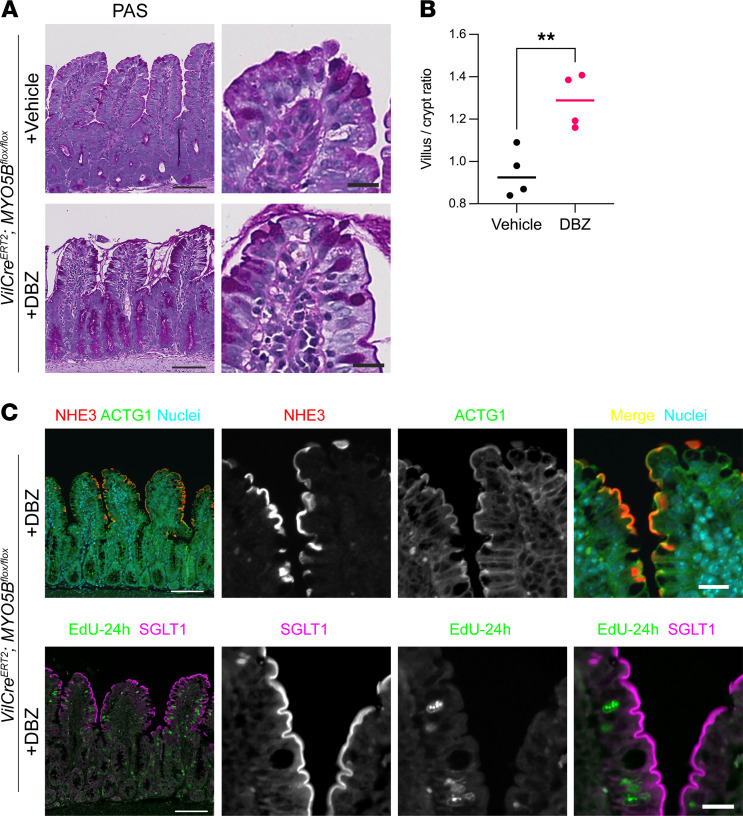
DBZ treatment improved enterocyte differentiation in tamoxifen-induced MYO5B-knockout mice. (**A**) PAS-stained jejunum of vehicle- or DBZ-treated *VilCre^ERT2^* *Myo5B^fl/fl^* mice. Paneth and goblet cells and brush borders were stained in dark purple. Scale bars: 100 and 10 μm. (**B**) Villus and crypt lengths were determined in 10 regions of jejunum of each mouse. Data point indicates the average value of individual mouse. *n* = 4 mice per group. **P < 0.01 by unpaired 2-tailed *t* test. (**C**) Brush border localization of NHE3 and SGLT1 in DBZ-treated MYO5B-deficient intestine. NHE3 was colocalized with the microvillus marker ACTG1 in a subset of enterocytes in villi. Some mice received EdU (5 mg/kg) 24 hours before euthanasia that was identified in some SGLT1^+^ villus cells, indicating that newly differentiated cells express SGLT1 with proper localization.

Hyperplasia of goblet cells and tuft cells induced by Notch inhibition has been identified in vivo and in organoid cultures (24–27). To test our hypothesis that MYO5B loss induces relatively high Notch with low Wnt signaling in intestinal epithelial cells, we next tested the effect of treatment with the Notch inhibitor dibenzazepine (DBZ) in MYO5B-knockout mice in vivo. Four days after the tamoxifen injection, *VilCre^ERT2^* *Myo5b^fl/fl^* mice typically had reduced body weight by approximately 15% and decreased mobility. Three days after the ip injection of DBZ (4 days after the tamoxifen injection), body weight loss in *VilCre^ERT2^* *Myo5b^fl/fl^* mice was not different compared to that in vehicle-treated *VilCre^ERT2^* *Myo5b^fl/fl^* mice. However, the mobility and signs of distress were improved by DBZ treatment (data not shown). As shown in Figure 8A, PAS-stained mucus in goblet cells and in the lumen was increased, and brush border structures were improved after DBZ treatment compared with vehicle-treated intestine. We have previously reported that MYO5B loss induces villus blunting, and 60%–70% of apical transporters are mislocalized away from their normal distribution in the brush borders (7). DBZ treatment significantly increased villus/crypt ratio in MYO5B-deficient intestine (Figure 8B), and their brush borders expressed sodium-proton exchanger 3 (NHE3) and sodium-dependent glucose transporter 1 (SGLT1) (Figure 8C). EdU^+^ enterocytes with proper localization of SGLT1 were identified in villus cells (Figure 8C), indicating that mature enterocytes were differentiated within 24 hours before the tissue collection. These observations indicate that DBZ inhibition stimulated enterocyte differentiation in *VilCre^ERT2^* *Myo5b^fl/fl^* mice.

**Figure 9:**
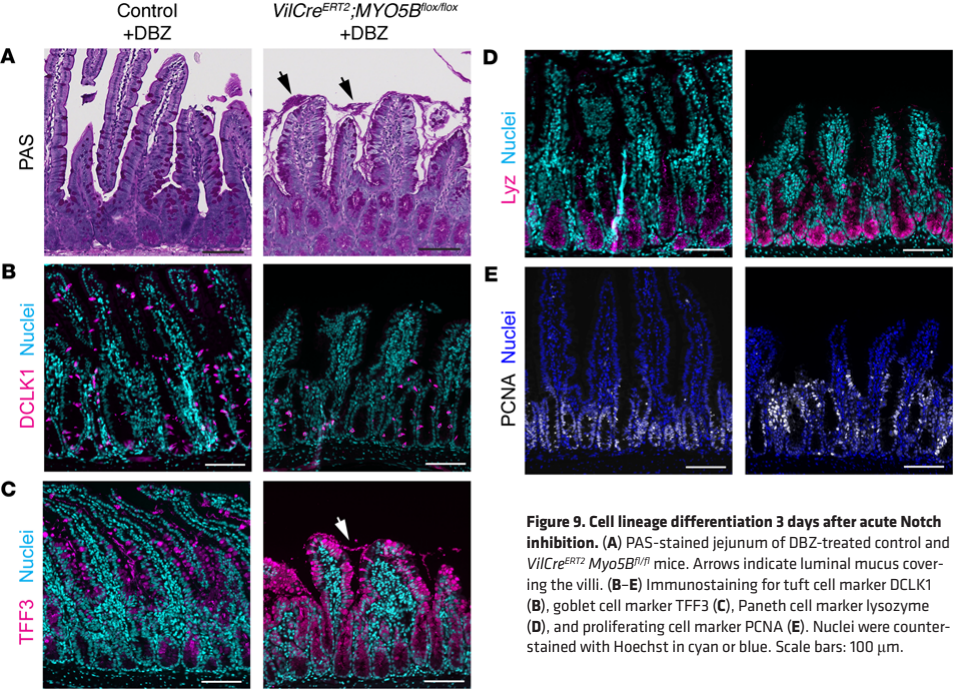
Cell lineage differentiation 3 days after acute Notch inhibition. (**A**) PAS-stained jejunum of DBZ-treated control and *VilCre^ERT2^* *Myo5B^fl/fl^* mice. Arrows indicate luminal mucus covering the villi. (**B**–**E**) Immunostaining for tuft cell marker DCLK1 (**B**), goblet cell marker TFF3 (**C**), Paneth cell marker lysozyme (**D**), and proliferating cell marker PCNA (**E**). Nuclei were counterstained with Hoechst in cyan or blue. Scale bars: 100 μm.

Consistent with the previous publications (27), DBZ-treated control mice in this study demonstrated increases in DCLK1^+^ tuft cells, TFF3^+^ goblet cells, and lysozyme^+^ Paneth cells as well as hyperproliferation that was detected by PCNA staining (Figure 9, left panels). Similarly, secretory cells were increased in *VilCre^ERT2^* *Myo5b^fl/fl^* mice after DBZ treatment (Figure 9, right panels), suggesting that Notch signaling is activated in MYO5B-deficient intestine.

## Discussion

In the present study, we quantified the frequency of differentiated secretory cells in tamoxifen-induced MYO5B-knockout mouse intestine to investigate the impact of MYO5B loss on epithelial cell lineage choice. As expected from our RNA-sequencing data, MYO5B loss significantly decreased tuft cells and goblet cells, whereas Paneth cells were increased together with expanded OLFM4-producing progenitor/stem cells in the crypts. Intestinal organoids recapitulated the reduction of tuft cells by MYO5B loss and showed that Wnt signaling may be involved in tuft cell differentiation. Systemic LPA administration specifically increased tuft cells both in control and MYO5B-deficient mouse intestines likely independent from Notch. Inhibition of Notch signaling accelerated secretory cell differentiation and partially normalized brush border structure, indicating Notch activity in MYO5B-deficient intestine. These observations suggest that MYO5B loss induces differentiation deficits in progenitor cells through Wnt/Notch imbalance and that LPA and Notch signaling can independently stimulate alternative pathways for epithelial differentiation.

MYO5B loss affected the differentiation of intestinal secretory cell lineages, including Paneth, goblet, enteroendocrine, and tuft cells. No significant change was detected in common transcription factors for those secretory lineages, such as *Math1*, *Atoh1*, *Gfi1*, or *Neurog3*, by RNA sequencing. Since some secretory cells live longer than 4 days, tamoxifen-induced MYO5B-knockout mice that survive only 4 days might not be an ideal model to investigate long-lived differentiated cells. Nevertheless, we found an 80% decrease in tuft cells, a 20% decrease in goblet cells, and an approximately 3-fold increase in Paneth cells in MYO5B-deficient intestine. Intriguingly, systemic LPA signaling specifically increased tuft cell frequency. The inhibition of tuft cell differentiation by MYO5B loss was consistent with that in organoid cultures. These observations indicate that tuft cell differentiation is likely upregulated by different pathways from other secretory cell lineages, which are dependent on a MYO5B-mediated mechanism and can be bypassed by LPA supplementation. To date, a number of tuft cell markers have been reported, including sensory molecules and secretory signal transmitters (28). Recent studies using multiplexed immunostaining and RNA sequencing following cell sorting techniques indicated that DCLK1^+^ tuft cells consist of many subsets that may serve different functions (17, 29, 30). A MYO5B-dependent and LPA-responsive subset of tuft cells might be different from naive tuft cells; however, further experiments are needed to identify them. We also demonstrated that ACTG1 immunostaining visualizes the distinct microvillus structure in tuft cells (Figure 1C), consistent with the observation in human intestine (11). Electron microscopic observations of intestinal tuft cells have revealed the long and wide microvilli, connected to a tubulovesicular network (31). Since the tuft cell microvilli lack LifeAct-GFP signal (unpublished observation), ACTG1 is a useful tool to investigate the tuft morphologies in paraffin sections. ACTG1 is 97% identical with cytoskeletal β-actin, but these 2 proteins are functionally distinguishable (32). ACTG1-deficient mice demonstrate deficits of mechanosensory stereocilia of hair cells, suggesting its distinct function in F-actin–based cytoskeletal stability (33). Although the existence of mechanosensory function in alimentary tuft cells is still controversial, ACTG1-mediated sensory function of tuft cells should be clarified in the future.

Gouyer et al. reported distinct goblet cell subpopulations along the crypt-to-villus axis that secrete fucosylated and nonfucosylated MUC2 throughout mouse ileum (18). TFF3 is an important factor for cytoprotection and is cosecreted with MUC2 in mouse intestine (34). The present results demonstrated a significant decrease in TFF3-producing goblet cells, and in particular, the UEA-1^–^ subset in MYO5B-deficient intestine (Figure 3). TFF3^+^UEA-1^+^ goblet cells were infrequently found in control intestine, while 50% of total goblet cells were dual positive in vehicle-treated MYO5B-deficient intestine (Figure 3C). This is likely due to incomplete maturation of goblet cells and maturation of MUC2 glycosylation resulting from MYO5B deficiency.

Paneth cells do not usually migrate toward the villus tip, and it is thought that the turnover of Paneth cells is longer than that of other types of differentiated intestinal cells. Lysozyme-positive granules were increased in MYO5B-deficient intestine, and Paneth cells were frequently found in the middle part of crypts within hyperproliferating progenitor cells (Figure 4). Despite the significant increase in Paneth cell frequency in MYO5B-knockout mice, RNA sequencing did not detect significant changes in transcription factors (*Klf15*, *Nlr4a1*) or Paneth cell–specific markers (*Lyz1*, *Ccrl2*, *Fzd9*, or *Darc*) (17). In addition, Paneth cell–derived Wnt3 transcription was significantly decreased by MYO5B loss (Figure 6A), suggesting that the maturation of Paneth cells might be inhibited in MYO5B-deficient intestine.

MYO5B loss positively and negatively changes the transcriptional signatures for stem and/or progenitor cells: *Lgr5*, *Ascl2*, and *Sp5* are downregulated, while *Tgif1*, *Mecom*, and *Arid5b* are significantly increased (7). The present study demonstrates remarkable expansion of OLFM4-expressing crypts that are specifically regulated by Notch signaling rather than Wnt (25). MYO5B-deficient epithelial cells expressed significantly lower levels of Wnt ligands (Figure 6A). Both Wnt and Notch activities are required to maintain stem cells of intestinal epithelial crypts, and a distinct Wnt/Notch combination defines each type of cell lineage differentiation (23). Although the lack of a good antibody against Lgr5 or LDL receptor related protein 6 (Lrp6) limits studies to confirm the protein localization of Wnt-dependent signaling pathways, our observations suggest that MYO5B loss induces Wnt/Notch imbalance and disrupts stem cell function to promote differentiation. Those alterations in stem/progenitor cells were not affected by LPA, consistent with the hyperproliferation and unchanged Notch target, HES1, indicating that LPA cannot normalize the abnormal stem cells and proliferating activities in MYO5B-knockout mice. Additionally, we confirmed the activity of Notch signaling in MYO5B-deficient intestine by using the Notch inhibitor DBZ in vivo. As previously reported, transient inhibition of Notch signaling induces OLFM4 reduction and hyperplasia of secretory cell lineages, including goblet and tuft cells (27), a phenotype opposite to that induced by MYO5B loss. *VilCre^ERT2^* *Myo5b^fl/fl^* mice similarly responded to DBZ treatment with an increase in secretory cells. More impressively, the differentiation of enterocytes expressing apical NHE3 and SGLT1 was enhanced by DBZ. These results suggest that MYO5B loss induces relatively low Wnt/high Notch imbalance that may cause cell differentiation deficits and that the modification of Notch signaling could be a therapeutic target.

Consistently, tuft cell differentiation was reduced by MYO5B loss in intestinal organoid cultures. DCLK1^+^ tuft cells were markedly fewer in undifferentiated organoids compared with differentiated organoids. MYO5B-deficient organoids demonstrated a similar number of tuft cells to undifferentiated organoids, suggesting that MYO5B loss directly inhibits differentiation of tuft cell lineage in progenitor cells. Inhibition of Notch signaling induces tuft cell hyperplasia in vivo and in vitro (25, 26). To our knowledge, however, the direct effect of Wnt signaling on tuft cell differentiation has not been investigated. In the present study, we found that the Wnt inhibitor, C59, in addition to the removal of exogenous Wnt in the differentiation media, significantly reduced tuft cell numbers in control organoids but did not affect MYO5B-deficient organoids (Figure 7C). The differences in tuft cell populations between control and MYO5B-iKO organoids were not as great as the difference in the intestinal tissues. It is likely because the Organoid Growth Medium (STEMCELL Technologies) contains a high amount of Wnt. Taken together with the decreased expression of Wnt ligands in MYO5B-deficient jejunum (Figure 6A), epithelial cell–derived Wnt ligands are likely depleted by MYO5B loss in organoid cultures. Those observations suggest that epithelial Wnt signaling likely potentiates the differentiation of tuft cells in mouse small intestine.

Our observations in the present study suggest that MYO5B is important for progenitor cell differentiation and that LPA signaling can stimulate an alternative pathway of tuft cell and enterocyte differentiation. The balance of Wnt/Notch signaling is likely impaired by MYO5B loss, and it may explain the alteration in secretory cell differentiation deficits. Further studies are needed to clarify precise cellular mechanisms of how MYO5B interacts with Wnt and Notch signaling molecules and which LPA receptor subtype(s) mediate the hyperplasia of tuft cells. Since the cellular mechanisms of LPA and Notch signaling likely generate overlapping influences on cell differentiation, rebalancing Wnt/Notch signaling may represent an effective therapeutic approach for treatment of MVID.

## Methods

### Mice.

*VilCre^ERT2^* and *Myo5b^fl/fl^* mice on a C57BL/6J background generated by our group (4) were separately maintained with more than 10 times backcrossing, and *VilCre^ERT2^* *Myo5b^fl/fl^* and littermate control mice were obtained by crossing those lines. Cre recombinase was activated at the age of 8–12 weeks with a single dose of tamoxifen (2 mg), as previously reported (4). LPA (3 mg/kg, 18:1 1-oleoyl-Lyso PA, 857231-P, Avanti Polar Lipids) solution was prepared and administered by og or ip injection once a day for 4 days as previously reported (7). Tamoxifen-injected *VilCre^ERT2^ Myo5b^fl/–^* or *Myo5b^fl/fl^* mice were used as controls. Some *VilCre^ERT2^* *Myo5b^fl/fl^* and *Myo5b^fl/fl^* (control) mice were ip injected with DBZ (30 μmol/kg, Tocris, Bio-Techne) (27) 1 day after tamoxifen injection. To trace proliferating cells, some mice received EdU (5 mg/kg) 24 hours before euthanasia. All mice were euthanized 4 days after tamoxifen injection, and the duodenum, jejunum, and ileum were collected.

### In vitro MYO5B-iKO organoids.

Intestinal organoids were generated from jejunal crypts of adult *VilCre^ERT2^ Myo5b^fl/fl^* mice and passaged twice with mechanical breakdown before the activation of Cre recombinase. One day after the second passage, 1 μM 4-OH-tamoxifen (SML1666, MilliporeSigma) was added into the Organoid Growth Medium (Mouse OGM, STEMCELL Technologies) and incubated for 24 hours (7). After the incubation with 4-OH-tamoxifen (iKO) or the vehicle, 100% ethanol (control), the medium was replaced with the modified Minigut medium (35) containing 5% Noggin-conditioned medium and 5% R-spondin–conditioned medium (Vanderbilt Organoid Core) to withdraw Wnt and EGF for enhancing cell differentiation.

### Immunohistochemistry.

Intestinal tissues were fixed with 10% neutral buffered formalin (NBF), embedded in paraffin as Swiss rolls, and cut into 4-μm-thin sections. Paraffin sections were deparaffinized in Histo-Clear (National Diagnostics) and a series of ethanol, then boiled under high pressure for 15 minutes in citrate buffer (S1699, Agilent). After preincubation with Protein Block (X0909, Agilent) for 1 hour, tissues were incubated with primary antibodies against DCLK1 (rabbit, 1:2000, ab109029, Abcam), TFF3 (rabbit, 1:1000, gift from Daniel K. Podolsky, the University of Texas Southwestern, Dallas, Texas, USA), lysozyme (rabbit, 1:400, ab108508, Abcam), ChgA (rabbit, 1:4000, 20085, ImmunoStar), OLFM4 (rabbit monoclonal, 1:200, D6Y5A, Cell Signaling Technology), MCT1 (guinea pig, 2 μg/mL; ref. 36), HES1 (rabbit, 1:200, 11988, Cell Signaling Technology), SGLT1 (rabbit, 0.5 μg/mL; ref. 7), and γ-actin (ACTG1; mouse monoclonal, sc-65638, Santa Cruz Biotechnology) that were diluted with Antibody Diluent with background reducing components (S3022, Agilent) for 1 hour at room temperature. Fluorescently labeled secondary antibodies (5 μg/mL, Jackson ImmunoResearch, 711-165-152, 711-175-152, 706-165-148, 715-606-151, 715-546-151) and FITC-conjugated UEA-1 (0.5 μg/mL, L9006, MilliporeSigma) were diluted with Antibody Diluent (S0809, Agilent) and incubated for 1 hour at room temperature. Nuclei were counterstained with Hoechst 33342 (4 mM, Thermo Fisher Scientific), diluted in PBS. EdU was visualized by Click-iT reaction from the Click-iT EdU Alexa Fluor 647 kit (C17340, Thermo Fisher Scientific). The stained slides were coverslipped with ProLong Gold Antifade Reagent (P36934, Thermo Fisher Scientific). Whole-slide fluorescence images were scanned by a Versa imager 200 with a 20× objective (Leica Biosystems) in the Vanderbilt Digital Histology Shared Resource (DHSR). *n* = 3–6 mice per group were analyzed.

For identifying goblet cells, coverslips were removed by heating slides after imaging of TFF3 and UEA-1 staining. The slides were rinsed in PBS, and PAS staining was performed at the Translational Pathology Shared Resource at Vanderbilt University Medical Center. The bright-field images were scanned by an SCN400 (Leica Biosystems).

### Whole-mount organoid staining.

Organoids were fixed with 10% NBF for 30 minutes in Matrigel domes, rinsed with cold PBS containing 1% BSA, and centrifuged at 600*g* for 1 minute at 4°C to remove Matrigel. Fixed organoids were blocked with 10% normal donkey serum in PBS containing 0.3% Triton X-100 for 2 hours and incubated with the DCLK1 antibody (1:4000) diluted with the blocking solution for 2 days at 4°C. After rinsing in PBS with Triton X-100, donkey anti-rabbit Cy3 antibody (2.5 μg/mL, 711-165-152, Jackson ImmunoResearch), Alexa Fluor 488 phalloidin (1:400, A12379, Thermo Fisher Scientific), and Hoechst 33342 (2 mM) were applied for 2 hours. Whole organoids and high-magnification images of native intestinal sections were imaged using a Nikon Ti-E microscope with an A1R laser scanning confocal system (Nikon Instruments Inc.).

### Digital image analysis.

The digital image analysis to quantify the frequency of differentiated cells was developed at the DHSR. Fluorescence images of individual markers were extracted as JP2 or TIF and tiled into small images to process in Cell Profiler (37). The whole pipeline of Cell Profiler that we built for cell identification is in Supplemental Data 1; supplemental material available online with this article; https://doi.org/10.1172/jci.insight.150416DS1 Total cell numbers were determined using Hoechst staining. For counting goblet (TFF3^+^), tuft (DCLK1^+^), or enteroendocrine (ChgA^+^) cells, immunoreactive cells, including nuclei, were identified and quantified using Cell Profiler scripts in whole small intestine. Paneth cells were identified by lysozyme immunostaining and green fluorescence signals without primary antibody in apical granules that were detected by the Spectrum Green filter cube (Chroma C169110; excitation 480, emission 535 nm).

### qRT-PCR.

Total RNA was prepared from isolated epithelial cells from jejunum of tamoxifen-induced or noninduced *VilCre^ERT2^ Myo5b^fl/fl^* mice by using TRIzol (Thermo Fisher Scientific), according to the manufacturer protocol. After reverse transcription of the RNA by using SuperScript III First-strand Synthesis System for RT-PCR (Thermo Fisher Scientific), cDNA was used as a template in PCR. Real-time quantitative PCR was performed utilizing SsoAdvanced Universal SYBR Green Supermix with CFX96 Real-Time System (Bio-Rad Laboratories). Previously published primer pairs were used (Supplemental Table 1). Target gene expressions were calculated relative to values from GAPDH by ΔΔCt method. RNA-Seq data that were generated in our previous study (7) (GEO database, accession number GSE139302) were analyzed for evaluating cell type–specific transcriptions.

### Statistics.

Statistical differences were determined using GraphPad Prism 9 with a *P* value of less than 0.05 considered significant. The test used in each analysis is described in the figure legends.

### Study approval.

The Institutional Animal Care and Use Committee of Vanderbilt University Medical Center, Nashville, Tennessee, USA, approved all experimental procedures and animal care.

## Author contributions

IK designed, performed, and analyzed studies and wrote the manuscript; JTR developed methods, analyzed data, and edited the manuscript; SRK, ACE, and AB performed experiments and edited the manuscript; AEG and MW provided essential materials; and JRG designed studies, provided research funds, and edited the manuscript.

## Supplementary Material

Supplemental table 1

Supplemental CellProfiler script

## Version 1 07/01/2021

In-Press Preview

## Version 2 08/23/2021

Electronic publication
