# Supplementary material for: Cell differentiation is disrupted by MYO5B loss through Wnt/Notch imbalance
Source: JCI Insight. 2021 Aug 23;6(16):e150416. doi: 10.1172/jci.insight.150416 (PMC8409988; doi:10.1172/jci.insight.150416)
Supplement: Supplemental table 1 [file jciinsight-6-150416-s107.pdf]

Supplemental Table 1. Primer sequences for qPCR

Gene	Forward	Reverse	
Wnt3	GGGGCGTATTCAAGTAGCTG	GTAGGGACCTCCCATTGGAT	(1)
Wnt6	TGCCCGAGGCGCAAGACTG	ATTGCAAACACGAAAGCTGTCTCTC	(2)
Wnt9b	AAGTACAGCACCAAGTTCCTCAGC	GAACAGCACAGGAGCCTGACAC	(2)
Dll1	CTGAGGTGTAAGATGGAAGCG	CAACTGTCCATAGTGCAATGG	(3)
Dll4	TCGTCGTCAGGGACAAGAATAGC	CTCGTCTGTTCGCCAAATCTTACC	(3)
Notch2	TGCCTGTTTGACAACTTTGAGT	GTGGTCTGCACAGTATTTGTCAT	(4)
Hes1	TGCCAGCTGATATAATGGAGAA	CCATGATAGGCTTTGATGACTTT	(4)
Gapdh	TTCCAGTATGACTCCACTCACGG	TGAAGACACCAGTAGACTCCACGAC	(5)

References

1. He W, et al. Wnt/beta-catenin signaling promotes renal interstitial fibrosis. *J Am Soc Nephrol.* 2009;20(4):765-76.
2. Davies PS, et al. Wnt-reporter expression pattern in the mouse intestine during homeostasis. *BMC Gastroenterology.* 2008;8(1):57.
3. Bohin N, et al. Rapid Crypt Cell Remodeling Regenerates the Intestinal Stem Cell Niche after Notch Inhibition. *Stem Cell Reports.* 2020;15(1):156-70.
4. Lim JS, et al. Intratumoural heterogeneity generated by Notch signalling promotes small-cell lung cancer. *Nature.* 2017;545(7654):360-4.
5. Ulatowski LM, et al. Strain-specific variants of the mouse Cftr promoter region reveal transcriptional regulatory elements. *Human Molecular Genetics.* 2004;13(17):1933-41.
